# Circulating MicroRNAs: Molecular Microsensors in Gastrointestinal Cancer

**DOI:** 10.3390/s120709349

**Published:** 2012-07-09

**Authors:** Moisés Blanco-Calvo, Lourdes Calvo, Angélica Figueroa, Mar Haz-Conde, Luis Antón-Aparicio, Manuel Valladares-Ayerbes

**Affiliations:** 1 Biomedical Research Institute of A Coruña, As Xubias 84, E-15006 A Coruña, Spain; E-Mails: Moises.Blanco.Calvo@sergas.es (M.B.-C.); Angelica.Figueroa.Conde-Valvis@sergas.es (A.F.); Maria.del.Mar.Haz.Conde@sergas.es (M.H.-C.); Luis.M.Anton.Aparicio@sergas.es (L.A.-A.); 2 Clinical Oncology Department, A Coruña University Hospital, As Xubias 84, E-15006 A Coruña, Spain; E-Mail: Lourdes.Calvo.Martinez@sergas.es; 3 Medicine Department, A Coruña University, Campus de Oza, E-15006 A Coruña, Spain

**Keywords:** circulating microRNAs, biomarkers, gastrointestinal cancer, oesophageal cancer, gastric cancer, pancreatic cancer, liver cancer, colorectal cancer

## Abstract

MicroRNAs (miRNAs) are small molecules of single strand non-coding RNAs, which are able to regulate gene expression. miRNAs have been involved in multiple cellular processes, such as proliferation, apoptosis and differentiation, thus alterations in miRNA expression have been shown to be directly linked with the pathological origin of multiple diseases, including cancer. In this way, during last few years, an increasing number of exciting advances have contributed to the understanding of miRNA roles in cancer. Moreover, researchers have exploited the special characteristics of miRNAs, such as the tissue and disease specificity or miRNA presence in blood, to explore their use as non-invasive tumour markers. In the present review, we summarize the current data on the potential usefulness of circulating miRNAs as diagnostic and prognostic tools in gastrointestinal tumours.

## 1. Introduction

Cancer is a major worldwide health issue and represents the second leading cause of death after cardiovascular diseases [1]. Among cancers, gastrointestinal (oesophageal, gastric, pancreatic, hepatic and colorectal) cancer is one of most diagnosed and, together with breast and lung tumours, is responsible for most deaths. Moreover, despite the intense efforts and advances in the understanding of the molecular causes of this disease, there is no definitive therapeutic solution. Current treatments have improved the curative expectations and the quality of life of patients; however, the effectiveness of these new tools depends largely on the stage in which tumours can be detected. Therefore, it is clearly needed to investigate potential biomarkers with capacity to detect malignancies at early stages and in a fast, simple, sensitive and specific way. While the detection of current cancer biomarkers is sufficiently fast and simple, unfortunately, their diagnostic and prognostic performance is poor, which hinders their clinical use [2]. Moreover, despite the large number of studies on circulating biomarkers for different tumours, few proposals have been translated into clinical practice.

MicroRNAs (miRNAs), small (18–22 nucleotides) single-stranded RNA molecules with regulatory functions [3], have become the focus of most recent efforts in cancer research. The importance of miRNAs lies in their extensive regulatory capacity, since a single miRNA is able to control the expression of hundred of genes [4,5], contributing to the global coordination of complex cellular processes, such as the proliferative control of stem cells [6]. Given this premise, the alteration in miRNA expression is considered one of the molecular abnormalities behind cancer development. In addition, miRNA expression is tissue specific [7] and therefore, the alteration of specific miRNAs in different tissues can be associated with concrete tumours [8]. In fact, it is possible to classify a tumour sample of unknown nature, even a metastatic one, by the identification of the tissue on which the primary tumour has been generated [9]. These characteristics make of miRNAs, powerful tools for diagnostic and prognostic purposes, as well as attractive therapeutic targets in cancer (for review, see [10]). Moreover, miRNAs are detected in blood at multiple physiological and pathological states, including cancer [11]. Most importantly, miRNAs are protected from degradation by ribonucleases in blood [12], enabling their detection and their use as non-invasive biomarkers. In this review, we summarize the current knowledge about the diagnostic and prognostic applications of circulating miRNAs in gastrointestinal cancer.

## 2. Sources of Circulating MiRNAs in Cancer

The origin and function of circulating nucleic acids in cancer, including miRNAs, is still under discussion (for a review, see [13]). The existence of circulating miRNAs in healthy individuals *per se* [14] or associated to different physiological events, such as pregnancy [15], underlines that their roles are not restricted to cancer. Therefore, circulating miRNAs in cancer may derive from multiple sources, including not only apoptosis and necrosis of circulating and primary tumour cells, but also the active release carried out by immune cells and other blood cells. Although there are different theories, the precise function of these circulating miRNAs in cancer remains unclear. One possibility is that cancer cells liberate immunosuppressive miRNAs allowing the tumour to evade the immune response. Cancer cells may also release oncogenic miRNAs contributing to their uncontrolled proliferation, the malignant transformation of surrounding cells and the recruitment of new blood vessels. Alternatively, immune cells may also produce miRNAs to stimulate the response against cancer cells while tumour surrounding cells may generate tumour-suppressive miRNAs to arrest the extension of malignancy (for review, see [16]).

Stability is another additional and interesting characteristic of circulating miRNAs which makes possible their detection and analysis, and, therefore, opening the door to their use as molecular markers. Moreover, this feature suggests that miRNAs do not circulate free in the bloodstream but are released as part of lipid or protein complexes that prevent the action of blood ribonucleases. The nature of these macromolecular complexes is in close relation to the source of transported miRNAs. Thus, while living cells actively release miRNAs encapsulated in large lipoprotein complexes (exosomes or microvesicles), miRNAs from dead or dying cells can be found in blood associated to Argonaute2 (Ago2) protein (for a review, see [10]). Since it has been hypothesised that most extracellular miRNAs, including plasma miRNAs, are part of Ago2 complexes, and given the increased amount of circulating miRNAs in cancer, it is possible speculate that these circulating miRNAs predominantly derive from apoptotic and necrotic processes occurring in tumour cells. Therefore, circulating miRNAs in cancer are a good reflection of the underlying disease, providing valuable tools to monitor the pathological changes during the clinical course of tumours. Taken together this premise and the miRNA tissue specificity, it can be proposed that circulating miRNAs are not only excellent biomarkers for cancer detection but also for prognostic purposes.

## 3. Circulating MiRNAs as Biomarkers in Gastrointestinal Cancer

In the last few years, studies on miRNAs as tumour markers have emerged as a field of special interest in gastrointestinal cancer according to the huge number of publications. However, while most studies are focused on the analysis of miRNA expression in tissue specimens, only a limited number of them have addressed the usefulness of circulating miRNAs as biomarkers in blood, serum or plasma samples. In this section, we will focus on the most relevant findings about the utility of circulating miRNAs as biomarkers in gastrointestinal cancer, *i.e.*, oesophageal, gastric, pancreatic, hepatic and colorectal cancer.

The first attempt to identify circulating miRNAs as biomarkers in oesophageal cancer (Table 1) was made by Zhang and co-workers in 2010 [17]. In this study, a set of seven miRNAs, including miR-10a, miR-22, miR-100, miR-148b, miR-223, miR-133a and miR-127-3p, was identified and validated as a diagnostic signature after analysing 290 serum samples from squamous cell carcinoma (SCC) patients. These seven miRNAs showed excellent diagnostic capacities in combination or as single markers, showing a great area under the Receiver Operating Characteristic (ROC) curves (AUCs). Although the AUC for the seven-miRNA signature was 0.929, the maximum value reached was for miR-22 serum determination (0.949) and the minimum value for miR-100 (0.817); both values are above the AUC (0.549) for the carcinoembryonic antigen (CEA), a conventional serum marker. In addition, the cluster analysis revealed the ability of seven-miRNA panel to discriminate between early stage patients and healthy subjects. In 2011, two additional reports focused on the diagnostic and prognostic roles of circulating miRNAs in oesophageal cancer were published simultaneously. The first one was focused on the analysis of miR-31 levels in serum from 201 SCC patients as a diagnostic and prognostic tool [18]. While the diagnostic performance of miR-31 was high both in the training (AUC = 0.902) and validation (AUC = 0.888) datasets, the most relevant conclusion was the independent association of high serum levels for miR-31 with poor relapse-free survival (hazard ratio: 3.260; 95% confidence interval: 1.264–8.421; *p*-value = 0.015). In the second study [19], an assay that combines the determination of miR-375 and miR-21 levels (miR-21/miR-375 ratio) in plasma for SCC diagnostic was developed, reaching an AUC of 0.816.

Regarding gastric cancer (Table 2), an incipient study published by Tsujiura and co-workers [20] evaluated by qRT-PCR the usefulness as diagnostic markers of five miRNAs (miR-17-5p, miR-21, miR-106a, miR-106b and let-7a) in plasma from 69 patients and 30 healthy individuals. The analysis of individual ROC curves demonstrated that miR-106b had the best AUC (0.721), while the combined assay for miR-106a/let-7a ratio improved this AUC, reaching 0.879. Therefore, miR-106b expression and miR-106a/let-7a ratio were proposed as the first non-invasive plasma biomarkers for gastric cancer diagnosis. MiR-106a was also detected in blood from gastric cancer patients and it was proposed to be a potential biomarker for detecting circulating tumour cells [21]. On the other hand, miR-17 alone or in combination with miR-106a, showed superior diagnostic properties as show the AUCs obtained from ROC curve analysis (0.684 for miR-106a, 0.743 for miR-17, and 0.741 for the combination) [21]. More recent and extensive studies investigated, by genome-wide approaches, circulating miRNAs with potential diagnostic applications in gastric cancer [22–24]. In one of these studies, Solexa sequencing was performed to discover miRNAs differentially expressed in pooled serum samples from 20 metastatic, 20 non-metastatic and 20 healthy donors. From this discovery stage, 22 miRNA candidates were selected and subsequently analysed by qRT-PCR in a training cohort of 22 gastric cancer cases and 22 controls. The five-miRNA signature (miR-1, miR-20a, miR-27a, miR-34 and miR-423-5p) thus identified was further validated by analysing serum from 142 gastric cancer patients and 105 matched controls. Both in training and validation datasets, the five-miRNA panel reached excellent diagnostic properties with respective AUCs of 0.879 and 0.831, clearly superior to AUCs obtained for the CA19-9 carbohydrate antigen and CEA (0.600 and 0.503, respectively) [22]. Other studies were performed using microarrays instead deep sequencing in the phase of biomarker discovery. Liu and co-workers [23] obtained seven miRNA candidates that were subsequently analysed by qRT-PCR in a training cohort of 30 gastric cancer patients and 30 healthy controls. Only three of them (miR-187*, miR-371-5p and miR-378) were selected for further validation in a cohort consisting of serum from 40 gastric cancer patients and 41 controls. The best AUC was obtained for miR-378 (0.861); in addition, a multivariate analysis, including the three candidate miRNAs, demonstrated that only miR-378 had diagnostic independence (odds ratio: 16.917; 95% confidence interval: 5.337–53.616; *p*-value < 0.0001) [23]. Microarrays were also used by Konishi and colleagues to identify miR-451 and miR-486 as plasma biomarkers for gastric cancer detection (respective AUCs: 0.96 and 0.92) [24]. However, Song and colleagues used qRT-PCR low-density arrays to identify miRNA biomarkers in serum of gastric cancer patients [25]. The panel of miRNAs obtained (miR-221, miR-376c, and miR-744) showed the best sensitivity (0.824) and specificity (0.588), using the cut-off values obtained from the analyses of ROC curves for each miRNA (AUCs: 0.70 for miR-221; 0.71 for miR-376c; and 0.74 for miR-744).

Finally, a recent study analysed the correlation between plasma miRNA levels and prognostic variables in gastric cancer [26]. They demonstrated that patients with elevated levels of miR-17-5p and miR-20a in plasma showed lower overall survival (*p*-value = 0.003 for both miRNAs). Moreover, as indicated by multivariate analysis, increased plasma levels of miR-20a was an independent risk factor for shorter overall survival in these patients (hazard ratio: 1.576; 95% confidence interval: 1.102–2.253; *p*-value = 0.013).

The first study on circulating miRNAs as biomarkers in pancreatic cancer patients was performed in 2009 by Wang and co-workers (Table 3) [27]. The authors analysed by qRT-PCR the expression of four miRNAs (miR-21, miR-210, miR-155 and miR-196a) in plasma from 49 pancreatic ductal adenocarcinoma (PDAC) patients and 36 healthy controls, achieving a good diagnostic performance for this miRNA panel. While the independent analysis of ROC curves for each miRNA gave rise to AUCs ranging from 0.62 (miR-21) to 0.69 (miR-196a), the combination of the entire panel improved the AUC (0.82) as well as the sensitivity (0.64) and specificity (0.89) at the optimal cut-point. In another posterior study, high levels of miR-196a in serum were associated with unresectable PDACs (AUC = 0.864) and shorter survival [28]. The same group analysed the expression of seven miRNAs (miR-16, miR-21, miR-155, miR-181a, miR-181b, miR-196a and miR-210) in plasma from pancreatic cancer patients, chronic pancreatitis patients and healthy individuals [29]. While the individual miRNAs reached good diagnostic properties, the combination of miR-16 and miR-196a provided the best results. The diagnostic capacity of this miRNA panel was further improved when it was combined with CA19-9 levels, increasing the AUC, specificity and sensitivity. Most importantly, the combinatory marker (miR-16 + miR-196a + CA19-9) showed an enhanced capacity for the detection of stage I pancreatic cancer compared to CA19-9 (respective detection rates: 85.2% and 55.6%). Two groups further explored the role of circulating miRNAs as diagnostic tools in pancreatic cancer. First, miR-18a was established as a powerful biomarker (AUC = 0.9369) for pancreatic cancer diagnostic in plasma [30]. Second, an extensive genome-wide analysis was performed by deep-sequencing in order to discover miRNAs with altered expression in serum from pancreatic cancer patients, followed by testing and validation of their potential diagnostic usefulness [31]. The analysis demonstrated the ability of a seven-miRNA signature (miR-20a, miR-21, miR-24, miR-25, miR-99a, miR-185, and miR-191) to discriminate pancreatic cancer from healthy controls (AUC = 0.992, *p*-value = 0.027 in training cohort; AUC = 0.985, *p*-value = 0.027 in validation cohort) and from chronic pancreatitis (AUC = 0.993; *p*-value = 0.008). More importantly, this seven-miRNA panel can be also used to detect pancreatic cancer cases at early stages (detection rates: 96.2% and 91.7% of cases at stages I and II), clearly improving the detection rates of conventional serum markers CA19-9 (detection rates: 46.2% and 62.5% of cases at stages I and II) and CEA (detection rates: 30.8% and 31.3% of cases at stages I and II). In addition, elevated levels of miR-21 in serum were identified as an independent predictor of poor survival in pancreatic cancer patients (Hazard ratio: 8.77; 95% confidence interval: 2.00–38.39; *p*-value < 0.01).

Up to date, most studies on liver cancer have focused on the most prevalent subtype, the hepatocellular carcinoma (HCC), and its close relationship with hepatitis B virus (HBV) infection and cirrhosis. This is also extensible to studies on circulating miRNAs and biomarker discovery (Table 4). In 2009, Yamamoto and co-workers reported the first work where significantly higher levels of miR-500 in serum of HCC patients were found, although diagnostic performance data were not provided [32]. Later on, for the first time, Gui and co-workers provided data on the diagnostic capacity of a circulating miRNA, miR-885-5p, in HCC. They obtained miR-885-5p from a global serum miRNA profiling using qRT-PCR low-density arrays. The AUC for the determination of miR-885-5p levels in serum was 0.904, which allowed to discriminate patients with different liver pathologies (HCC, chirrosis, and chronic hepatitis B) from healthy controls with a sensitivity of 90.53% and a specificity of 79.17% [33]. A more comprehensive study was conducted by using deep-sequencing technology, achieving the identification of two miRNA signatures in serum that allow the diagnosis of hepatitis B/C infection and hepatitis B virus (HBV)-associated HCC [34]. The miRNA signature for HBV-associated HCC diagnosis was composed of miR-25, miR-375, and let-7f. These miRNAs were able to discriminate cases from healthy controls with excellent specificity and sensitivity (99.1% and 97.9%, respectively; AUC: 0.9967). Interestingly, a single component of the previous signature, miR-375, maintains exceptional diagnostic parameters with a specificity of 96% and a sensitivity of 100%. In a similar way, two miRNAs, miR-10a and miR-125b, were capable to distinguish HBV-infected patients from HBV-associated HCC cases with a specificity and sensitivity of 98.5%, and an AUC of 0.992 [34]. A later work performed also on serum samples allowed the discovery of miR-21, miR-122 and miR-223 as excellent biomarkers for HCC (respective AUCs = 0.87, 0.79 and 0.86) and chronic hepatitis (respective AUCs = 0.91, 0.93 and 0.88) [35]. Moreover [36], on the basis of previous works, seven miRNAs (miR-1, miR-16, miR-122a, miR-139, miR-195, miR-199a, and miR-224) were evaluated in serum with the purpose to discriminate HCC from chronic liver diseases. Since miR-1, miR-122a, miR-139, and miR-224 were undetectable in serum, the diagnostic reliability for HCC detection was determined for the remaining miRNAs (miR-16, miR-199a, and miR-195). From these three miRNAs, only miR-16 and miR-199a showed capacity to separate patients with HCC from those with chronic liver disease. By using the cut-off values provided by the distribution curves of each miRNA, both miRNAs showed superior sensitivity than conventional serum markers (AFP: α-fetoprotein; AFP-L3: *Lens culinaris* agglutinin-reactive α-fetoprotein; DCP: des-γ-carboxyprothrombin) to separate HCC and chronic liver diseases. The best sensitivity (92.4%) and specificity (78.5%) was achieved by the combination of miR-16 with conventional serum markers. MiR-16 used in second-line testing, after conventional serum markers, was also able to detect HCC in 69.2% of initially negative cases. By a genome-wide exploratory approach, similar to the procedure used by Lee and co-workers in a previous work [34] but based on microarray analysis instead of deep sequencing, plasma miRNAs were recently identified as biomarkers for HBV-related HCC diagnosis [37]. Seven plasma miRNAs (miR-122, miR-192, miR-21, miR-223, miR-26a, miR-27a, and miR-801) were proposed as diagnostic signature with capacity to discriminate HBV-related HCC from healthy controls (specificity: 93.9%; sensitivity: 83.2%; AUC: 0.941) and hepatitis B (specificity: 79.1%; sensitivity: 76.4%; AUC: 0.842). Although he components of this miRNA panel do not match to any of the previously employed by Li and co-workers [34], in other studies [35], however, they have used several of these miRNAs. This is the case of miR-122, which was again identified as serum biomarker to distinguish HBV-related HCC patients from healthy controls (AUC = 0.869) in the Chinese population [38]. Finally, only one study assessed the usefulness of circulating miRNAs as prognostic tools in HCC [39]. In this study, elevated serum levels of miR-221 were associated to shorter overall survival (*p*-value < 0.05) and identified as an independent risk factor for poor prognosis in HCC patients (Hazard ratio = 1.903; 95% confidence interval = 1.235–2.981; *p*-value = 0.018) [39].

Attending to circulating miRNAs in colorectal cancer (Table 5), Chen and colleagues [14] conducted for the first time a comprehensive analysis of expression profile of miRNAs in serum by deep sequencing. They detected several miRNAs differentially expressed in colorectal cancer compared to those found in healthy subjects and lung cancer patients. However, Ng and co-workers provided data about the diagnostic performance of miRNAs in plasma of colorectal cancer patients [40]. In this study, by analysing 95 miRNAs in plasma form 130 patients and 50 healthy controls, miR-17-3p and miR-92 were demonstrated to be the best tools to detect colorectal cancer with respective AUCs of 0.717 and 0.885. Later on, miR-92 (now renamed as miR-92a) and miR-29a, also demonstrated their capacity, independently or in combination, to successfully discriminate plasma samples from colorectal cancer patients, patients with advanced adenomas and healthy controls [41]. When they were used in combination, miR-92a and miR-29a plasma levels showed AUCs of 0.883 and 0.773 to detect respectively colorectal cancer and adenomas from healthy subjects. MiR-29a levels in serum were also used to detect liver metastasis in colorectal cancer patients with an AUC of 0.803, reaching a sensitivity and specificity of 0.75 [42]. In relation to prognosis, the abundance of miR-141 in plasma was associated with stage IV colon cancer and it displayed an elevated ability to diagnose this subset of patients. This ability is complemented and enhanced when the miR-141 assay is combined with CEA determination. Moreover, high levels of miR-141 in plasma were related to poorer survival in colon cancer patients, independently of tumour stage, gender, and age [44]. Likewise, elevated levels of miR-221 in plasma were also proposed as an independent prognostic factor for overall survival in colorectal cancer patients (hazard ratio: 3.478; 95% confidence interval: 1.038–11.654; *p*-value = 0.043) [43]. However, the AUC (0.606) obtained for miR-221 when was used to detect colorectal cancer from healthy controls, was modest in comparison to other circulating miRNA investigated [44].

## 4. Conclusions

To date, since the discovery of miRNAs as regulatory molecules, a large number of reports have elucidated their function, the mechanisms of action, and their expression profile in a wide spectrum of organisms, samples and conditions. This huge amount of information has led researchers to understand the importance of miRNAs as central players during both the initiation and clinical development of many diseases, including cancer. Beyond the possible therapeutic use, these findings have impelled the research on miRNAs as clinical biomarkers. In fact, the singular characteristics of miRNAs, such as tissue- and disease-specificity, make their use an excellent option to detect physiological and pathological alterations. Thus, miRNAs are able to identify, not only the presence or absence of tumours, but also can determine the primary organ or tissue affected and the clinical and pathological stage. Furthermore, miRNAs can predict risk of progression, relapse, and metastasis, and help to evaluate possible clinical scenarios in relation to the therapy response.

Moreover, because of their presence and stability in different body fluids [45], the primary future application of miRNAs is their utilization as non-invasive markers. However, the promising findings performed to date are hampered by the variability in sample sources. In addition to the determination of miRNAs in blood, they have been also successfully isolated from clinically relevant samples, such as stool [46] and urine [47], and from forensic samples, such as saliva and semen [48]. However, the favourite sample type is blood derivative due to the reliable determination of the disease status in these samples and the presence of higher amounts of miRNAs. Among blood derivatives, serum is most frequently used in biomarker studies, probably to avoid the technical issues related to the presence of anticoagulants in plasma. Up to now, no relevant studies have been performed on whole blood, due to the possible interference of miRNAs from blood cells. However, the use of whole blood to search for miRNA biomarkers could be interesting as, conversely to plasma and serum, handling is not necessary prior obtaining the sample. Consequently, this sample type provides a scalable solution for future technological implementations for fast and *in situ* determinations.

Therefore, due to the obstacles related to heterogeneity of available detection techniques and sample sources, there are few clinical trials conducted to evaluate the potential clinical application of miRNA biomarkers. However, the mentioned impediments will be undoubtedly overcome and the clinical implementation of circulating miRNAs as genuine “microsensors” in cancer will become a reality in the near future.

## Figures and Tables

**Table 1.** Circulating miRNAs with diagnostic and prognostic applications in oesophageal cancer.

**miRNAs**	**Samples (n)**	**TNM** ^1^ **Stage (patients)**	**Technique**	**Up/ Down** ^2^	**Association**	**Ref.** ^3^
7-miRNA panel: miR-10a, miR-22, miR-100, miR-133a, miR-127-3p, miR-148b, miR-223	Serum (290 patients, 140 controls)	0 (2), I (18), II (81), III (31), IV (11), X ^6^ (6)	Deep-sequencing + qRT-PCR ^4^	Up	Diagnosis of SCC	[17]
miR-31	Serum (201 patients, 202 controls)	I (28), II (31), III (33), IV (28)	qRT-PCR	Up	Poor relapse-free and tumour-specific survival/Diagnosis of SCC	[18]
miR-21/ miR-375 ratio	Plasma (50 patients, 20 controls)	I-II (25), III-IV (25)	qRT-PCR	Up	Diagnosis of SCC ^5^	[19]

1 Tumor, Node, Metastasis staging system;

2 Up-regulation or Down-regulation regarding control samples;

3 References;

4 Quantitative reverse transcription polymerase chain reaction;

5 Squamous cell carcinoma;

6 Unknown.

**Table 2.** Circulating miRNAs with diagnostic and prognostic applications in gastric cancer.

**miRNAs**	**Samples (n)**	**TNM** ^1^ **Stage (patients)**	**Technique**	**Up/ Down** ^2^	**Association**	**Ref.** ^3^
miR-106b	Plasma (69 patients, 30 controls)	I (38), II (13), III (14), IV (4)	qRT-PCR ^4^	Up	Gastric cancer diagnosis	[20]
miR-106a/ let-7a ratio	Plasma (69 patients, 30 controls)	I (38), II (13), III (14), IV (4)	qRT-PCR	Up	Gastric cancer diagnosis	[20]
miR-17, miR-106a	Blood (90 patients, 27 healthy controls)	Not provided	qRT-PCR	Up	Circulating tumour cell detection; gastric cancer diagnosis	[21]
5-miRNA signature: miR-1, miR-20a, miR-27a, miR-34, miR-423-5p	Serum (164 patients, 127 controls)	I (29), II (56), III (48), IV (23), X ^5^ (8)	Deep sequencing + qRT-PCR	Up	Gastric cancer diagnosis	[22]
miR-378	Serum (57 patients, 61 controls)	I (6), II (14), III (18), IV (19)	Microarray + qRT-PCR	Up	Gastric cancer diagnosis	[23]
miR-451, miR-486	Plasma (56 patients, 30 controls)	I+II (33), III-IV (23)	Microarray + qRT-PCR	Up	Gastric cancer diagnosis	[24]
miR-221, miR-376c, miR-744	Serum (82 patients, 82 controls)	Not provided	qRT-PCR array	Up	Gastric cancer diagnosis	[25]
miR-17-5p, miR-20a	Plasma (87 patients)	I+II (40), III (37), IV (10)	qRT-PCR	Up	Poorer overall survival	[26]

1 Tumor, Node, Metastasis staging system;

2 Regarding control samples;

3 References;

4 Quantitative reverse transcription polymerase chain reaction;

5 Unknown.

**Table 3.** Circulating miRNAs with diagnostic and prognostic applications in pancreatic cancer.

**miRNAs**	**Samples (n)**	**Stage (patients)**	**Technique**	**Up/ Down** ^1^	**Association**	**Ref.** ^2^
4-miRNA signature: miR-21, miR-210, miR-155, miR-196a	Plasma (49 patients, 36 controls)	Localized (15), locally advanced (13), metastatic (21)	qRT-PCR ^3^	Up	Diagnosis of pancreatic adenocarcinoma	[27]
miR-196a	Serum (35 patients, 15 pancreatitis, 15 controls)	TNM ^4^: IB (9), IIA (6), IIB (9), III (5), IV (6)	qRT-PCR	Up	Un-resectability and short survival	[28]
2-miRNA signature: miR-16, miR-196a (±CA^5^19-9)	Plasma (138 tumours, 107 pancreatitis, 68 controls)	TNM: I (27), II (39), III (17), IV (55)	qRT-PCR	Up	Early diagnosis of pancreatic cancer; chronic pancreatitis diagnosis	[29]
miR-18a	Plasma (36 patients, 30 controls)	TNM: Ib (1), IIa (7), IIb (13), IV (8)	qRT-PCR	Up	Diagnosis of pancreatic cancer	[30]
7-miRNA panel: miR-20a, miR-21, miR-24, miR-25, miR-99a, miR-185, miR-191	Serum (197 tumours, 82 pancreatitis, 158 healthy donors)	TNM: I (26), II (48), III (45), IV (66), X ^6^ (12)	Deep sequencing + qRT-PCR	Up	Early diagnosis of pancreatic cancer	[31]
miR-21	Serum (38 tumours)	Not provided	qRT-PCR	Up	Poor survival	[31]

1 Regarding control samples;

2 References;

3 Quantitative reverse transcription polymerase chain reaction;

4 Tumor, Node, Metastasis staging system;

5 Carbohydrate antigen;

6 Unknown.

**Table 4.** Circulating miRNAs with diagnostic and prognostic applications in HCC ^1^.

**miRNAs**	**Samples (n)**	**Stage (patients)**	**Technique**	**Up/ Down** ^2^	**Association**	**Ref.** ^3^
miR-885-5p	Serum (46 tumours, 15 liver diseases, 26 cirrhosis, 23 HBV, 24 controls)	Not provided	qRT-PCR ^4^ array	Up	Diagnosis of liver pathologies	[33]
3-miRNA signature: miR-25, miR-375, let-7f	Serum (55 patients, 50 controls)	Not provided	Deep sequencing + qRT-PCR	Up	Diagnosis of HBV^5^-related HCC	[34]
miR-21, miR-122, miR-223	Serum (101 tumours, 48 HBV, 89 healthy controls)	Not provided	qRT-PCR	Up	Detection of HCC and/or HBV (liver injury)	[35]
miR-16 (+/− conventional serum markers: AFP ^6^, AFP-L3 ^7^, DCP ^8^)	Serum (105 tumours, 107 chronic liver diseases, 71 controls)	CLIP ^9^: 0-2 (48), 3-6 (15)	qRT-PCR	Down	HCC detection in combination or in second-line after testing conventional serum makers	[36]
7-miRNA combination:miR-21, miR-26a, miR-27a, miR-122, miR-192, miR-223, miR-801	Plasma (400 tumours, 116 cirrhosis, 147 HBV, 134 healthy donors)	BCLC ^10^: 0 (62), A (243), B (69), C (25), D (1)	Microarray + qRT-PCR	Up/ Down	Diagnosis of HBV-related HCC	[37]
miR-122	Serum (72 tumours, 48 HBV, 34 healthy donors)	TNM ^11^: I (8), II (36), III (20), IV (6)	qRT-PCR	Up	Detection of HBV-related HCC	[38]
miR-221	Serum (46 tumours, 20 controls)	TNM: I (16), II (19), III-IV (11)	qRT-PCR	Up	Shorter overall survival	[39]

1 Hepatocellular carcinoma;

2 Regarding control samples;

3 References;

4 Quantitative reverse transcription polymerase chain reaction;

5 Hepatitis B virus;

6 α-fetoprotein;

7 *Lens culinaris* agglutinin-reactive α-fetoprotein;

8 des-γ-carboxyprothrombin;

9 CLIP (Cancer of the Liver Italian Program) staging system;

10 Barcelona Clinic Liver Cancer staging system;

11 Tumor, Node, Metastasis staging system.

**Table 5.** Circulating miRNAs with diagnostic and prognostic applications in colorectal cancer.

**miRNAs**	**Samples (n)**	**TNM** ^1^ **Stage (patients)**	**Technique**	**Up/ Down** ^2^	**Association**	**Ref.** ^3^
miR-17-3p, miR-92	Plasma (130 CRC patients, 50 healthy controls)	I (6), II (35), III (39), IV (50)	qRT-PCR ^4^	Up	Colorectal cancer diagnosis	[40]
miR-29a, miR-92a	Plasma (100 CRC patients, 37 adenomas, 59 healthy controls)	I (27), II (25), III (38), IV(10)	qRT-PCR	Up	Colorectal cancer and adenoma diagnosis	[41]
miR-29a	Serum (114 patients)	Not provided	qRT-PCR	Up	Liver metastasis detection	[42]
miR-141 (+CEA ^5^)	Plasma (185 CRC patients, 76 healthy controls)	I (11), II (63), III (49), IV (62)	qRT-PCR	Up	Poor survival	[43]
miR-221	Plasma (103 patients, 37 healthy controls)	I-II (36), III-IV (44)	qRT-PCR	Up	Colorectal cancer diagnosis; poor overall survival	[44]

1 Tumor, Node, Metastasis staging system;

2 Regarding control samples;

3 References;

4 Quantitative reverse transcription polymerase chain reaction;

5 Carcinoembryonic antigen.
